# Diminishing Hepcidin via Reducing *IL-6*/STAT3 Pathway by Utilizing Ferulic Acid: An In Vitro Study

**DOI:** 10.3390/biomedicines13040923

**Published:** 2025-04-09

**Authors:** Ola M. Al-Sanabra, Luay F. Abu-Qatouseh, Mohammad I. A. Ahmad, Mutaz Jamal Al-Khreisat, Majd M. Alsaleh

**Affiliations:** 1 Department of Medical Laboratory Sciences, Faculty of Allied Medical Sciences, Al-Balqa Applied University, Al-Salt 19117, Jordan; 2 Department of Medical Analyses, Faculty of Pharmacy and Medical Sciences, University of Petra, Amman 11196, Jordan; labuqatouseh@uop.edu.jo; 3 Department of Medical Laboratory Sciences, Faculty of Allied Medical Sciences, Al-Ahliyya Amman University, Amman 19328, Jordan; m.malkawy@ammanu.edu.jo (M.I.A.A.); m.alkhreisat@ammanu.edu.jo (M.J.A.-K.); 4 Pharmacological and Diagnostic Research Center (PDRC), Faculty of Pharmacy, Al-Ahliyya Amman University, Amman, 19328, Jordan; 5 Department of Allied Medical Sciences, Al-Balqa Applied University, Al-Salt 19117, Jordan; majd.alsaleh@bau.edu.jo; 6 Department of Biology, Faculty of Science, University of Jordan, Amman 11942, Jordan

**Keywords:** hepcidin, *IL-6*/STAT3 pathway, ferulic acid, hypoferremia, anemia

## Abstract

**Background/Objectives**: Hepcidin is a negative regulator of iron absorption that is released by hepatocytes. It is one of the main contributors to hypoferremia and anemia in inflammatory and oncological disorders that are mediated by the proinflammatory cytokine *IL-6*/STAT3 pathway. Ferulic acid (FA) is a phenolic compound with pleiotropic biological activities, including anti-inflammatory activity. However, its effect on hepcidin secretion is still unknown. Thus, this study aimed to explore the impact of FA on hepcidin levels and the underlying mechanism. **Methods**: HepG2 cells were treated with different log percentages of FA, and their viability was determined via the MTT assay. The relative expression of *IL-6* and *HAMP* in treated and untreated cells was measured via qRT-PCR, and the protein levels of hepcidin, *IL-6* and STAT3 were measured using ELISA. **Results**: The MTT test showed an inverse relationship between FA concentrations and HepG2 cell proliferation; FA’s IC_50_ value was 0.07669%. The expression levels of *IL-6* and *HAMP* were significantly increased in HepG2 cells following 24 h of culture with 4 μg/mL LPS. Meanwhile, the addition of FA significantly decreased the relative expression levels of these two genes and the secretion levels of *IL-6*, STAT3 and hepcidin compared to the cells treated with LPS alone. **Conclusions**: Overall, these findings show that FA modifies inflammatory pathways, affecting hepcidin levels via the *IL-6*/STAT3 pathway. Thus, this suggests FA as a potential therapeutic agent against the hypoferremia and anemia developed due to dysregulated hepcidin levels in diseases such as inflammatory and oncological disorders.

## 1. Introduction

Iron homeostasis is a critical physiological process that ensures the balance of iron levels in the body and prevents both iron deficiency and overload. The typical serum iron concentration in adults ranges from 60 to 200 μg/100 mL, and variations beyond this range have been observed in healthy individuals, indicating a potential variability in iron regulation [1]. Central to this regulation is hepcidin, a liver-derived hormone that responds to changes in iron levels and inflammatory signals. Elevated iron stores or reduced erythropoietic activity stimulates hepcidin production, while conditions such as anemia and hypoxia suppress it [2]. Hepcidin regulates iron levels by binding to ferroportin, the primary cellular iron exporter, which leads to its internalization and degradation. This process, which occurs in the spleen, duodenum and placenta, reduces dietary iron absorption and iron release from cells, increasing intracellular iron stores and decreasing levels of circulating iron [3]. Inflammation significantly influences hepcidin production, with proinflammatory cytokines such as interlukein-6 (*IL-6*) playing a key role [4]. *IL-6* stimulates hepcidin synthesis in hepatocytes via the JAK2-STAT3 signaling pathway, which regulates hepcidin expression from the *HAMP* gene located on chromosome 19 [5,6].

Cancer has increasingly begun to be recognized as a metabolic disorder with inflammation playing a pivotal role in its initiation and progression. Metabolic alterations in cancer cells, including changes in glucose, fatty acids, glutamine, and iron metabolism, are driven by oncogenes and tumor suppressor genes. These changes not only meet the heightened energy demands of cancer cells but also create an inflammatory tumor microenvironment that promotes tumor proliferation and metastasis [7]. Iron, which is essential for DNA synthesis and repair [8], is often deregulated in cancer, leading to iron overload and the disruption of homeostasis. Elevated hepcidin levels are associated with poor survival outcomes in cancer patients, particularly during hematopoietic stem cell transplantation [9], and have been observed in various cancers, including colorectal carcinoma [10], breast carcinoma [11], esophageal carcinoma [12], urothelial and renal neoplasms [13] and hepatic carcinoma [14]. This suggests that hepcidin may serve as a prognostic biomarker in cancer.

Ferulic acid (FA), a natural compound with well-documented antioxidant and anti-inflammatory properties, has shown promise in mitigating the inflammation associated with hepatocellular carcinoma (HCC), the most common liver cancer [15]. Chronic liver injury and inflammation are the primary drivers of HCC, with inflammatory signaling pathways facilitating the transition from liver damage to malignancy [16]. Cytokines, which are critical mediators of inflammation, are implicated in HCC development, highlighting the importance of targeting these pathways in therapeutic interventions [17]. FA has been shown to modulate cytokine production, potentially alleviating the inflammatory environment that promotes liver cancer progression [18]. Furthermore, iron homeostasis, which is governed by hepcidin, is frequently disrupted in chronic liver disease and inflammation, thereby complicating the pathophysiology of HCC [16]. Given the frequent disruption of iron homeostasis in these conditions, the interplay between FA’s anti-inflammatory effects and hepcidin regulation offers a promising strategy for reducing liver cancer risk. Investigating the synergistic effects of the modulation of FA and hepcidin could provide novel insights into therapeutic approaches for liver cancer prevention and management [16].

Despite FA’s potential in treating inflammatory and oncological disorders, the complexity of liver diseases and the multifactorial nature of cancer necessitate further research. A deeper understanding of FA’s role in iron homeostasis and its interaction with hepcidin is essential to determine its efficacy and safety in clinical applications. This study aimed to explore the impact of FA on hepcidin levels in vitro, marking the first investigation of the connection between FA and hepcidin modulation. By investigating this relationship, we hope to contribute to the development of innovative therapeutic strategies for liver cancer and iron-associated disorders.

## 2. Materials and Methods

### 2.1. Chemical Reagents and Ferulic Acid (FA) Preparation

A stock of FA solution was prepared by dissolving (100 mg) FA with purity > 98% (Abcam, Cambridge, UK) in 1 mL of dimethyl sulfoxide (DMSO) with purity > 99% (ThermoFisher, Waltham, MA, USA). Afterwards, different log percentages of FA were prepared by diluting the stock solution.

### 2.2. Cell Line and Culture Conditions

The hepatocellular carcinoma-derived cell line HepG2 (HB-8065) was purchased from ATCC, Washington, DC, USA, and was cultured in modified Eagle medium (PAN-Biotech, GmbH, Aidenbach, Germany) supplemented with 10% fetal bovine serum (FBS) (Cytiva, Amersham, UK), 1% L-Glutamine (PAN-Biotech, GmbH), 1% penicillin–streptomycin (Capricorn Scientific GmbH, Ebsdorfergrund, Germany), 1% Amphotericin-B (PAN-Biotech, GmbH), 1% nonessential amino acids (EuroClone, Pero, Italy) and 1% sodium pyruvate (EuroClone) in a humified atmosphere of 5% of CO_2_ and 37 °C. To ensure that the cell line was established, the HepG2 cells were passaged three times before starting the experiments. The medium was changed on alternate days to maintain optimal cell growth conditions.

### 2.3. Cell Viability Assessment

When the cell confluency was 70–80% in the fourth passage, the cells were detached via addition of 0.05% trypsin/EDTA (PAN-Biotech, GmbH). A total of 7 × 10^3^ HepG2 cells were cultured in each well of a 96-well culture plate (SPL Life Science Co., Ltd., Pocheon-si, Republic of Korea), and the plate was incubated under the same culturing conditions mentioned previously. The seeded cells were treated with different log percentages of FA (−0.02–−0.5%) or vehicle (0.2%) DMSO and incubated for 24 h. Viability was examined via the MTT assay (Sigma, St. Louis, MO, USA). Briefly, after incubation for 24 h, 5 mg/mL of MTT reagent assay was added to the treated cells, and the plate was incubated for 3 h under similar culturing conditions. Next, as an indicator of the cell viability, the intensity and change in the reagent color in the plate were measured using a Multiskan GO spectrophotometer (Thermo Scientific, Cambridge, UK) at a wavelength of 570 nm. The cell viability assessment was conducted in triplicate.

### 2.4. Cell Treatment

At a concentration of 1 × 10^6^, HepG2 cells were incubated in six-well plates containing the MEM with 10% FBS were incubated under a humified 5% CO_2_ atmosphere at 37 °C for 24–48 h. Next, the medium was aspirated from each well and the following treatments were applied: fresh media, 0.1% stock FA only, 0.01% DMSO, 4 µg lipopolysaccharide (LPS from *E. coli O55:B5*, ChemCruz, London, UK), 0.1% stock FA + 4 µg LPS. The plates were then incubated for an additional 24 h. Afterwards, the cells were trypsinized and harvested for either the extraction and isolation of RNA or for STAT3 levels’ measurement. The HepG2 cell treatment was performed in triplicate.

### 2.5. RNA Extraction, cDNA Synthesis, and qRT-PCR

Total RNA was extracted and isolated from the harvested cells using the EasyPure RNA Kit (Transgen Biotech, Beijing, China) as per the manufacturer’s instructions. Purity assessment and quantification of the isolated RNA were performed using a µDrop plate and Multiskan GO spectrophotometer (Thermo Scientific) at 260 and 280 nm. Then, 70 ng of total RNA was reverse transcribed to cDNA using the EasyScript First-Strand cDNA synthesis SuperMix kit (Transgen Biotech) following the manufacturer’s protocol. Next, the qPCR reaction was carried out by using AriaDx Real-Time PCR (Diatech, Jesi, Italy) and the real-time SYBR Green method. Briefly, the 20 µL of amplification reaction consisted of 1 µL of cDNA, 10 µL of PerfectStart Green qPCR SuperMix (Transgen Biotech), 0.4 µL of 50× Universal Passive Reference Dye, 0.8 µL of 10 µM primers (IDT, Coralville, IA, USA) and 7.8 µL of water. The reaction was performed starting with initial inactivation at 94 °C for 30 s, followed by 40 cycles of denaturation at 94 °C for 5 s, annealing at 60 °C for 30 s and, finally, melting at 94 °C for 1 min. The relative mRNA expression level was determined using *GAPHD* as a housekeeping gene following the comparative 2^−ΔΔCT^ calculation formula. The sequences of the primers used are shown in Table 1.

### 2.6. Quantification of IL-6, STAT3 and Hepcidin Levels

The culture supernatants from the incubated untreated and treated (LPS only, FA only and LPS + FA) cells were collected after 24 h by centrifugation at 3000 rpm for 20 min. Subsequently, commercial ELISA kits were used to measure the levels of secreted *IL-6* (Invitrogen ThermoFisher Scientific) and hepcidin (Sunlong Biotech Co., Ltd., Hangzhou, China) following the manufacturer’s instructions. For measuring phospho-STAT3, untreated and treated cell lysates were assessed using a commercial ELISA kit (R&D, Minneapolis, MN, USA) as per the manufacturer’s instructions on the protocol.

### 2.7. Statistical Analysis

Data are presented as mean ± standard deviation (M ± SD). Outlier removal and checks for normality of residuals were performed before statistical comparisons. Statistical analysis was performed via one-way analysis of variance (ANOVA) or Student’s paired *t*-test using GraphPad Prism Software 8 (GraphPad Software Inc., La Jolla, CA, USA).

## 3. Results

### 3.1. Determination of the IC_50_ and Viability of HepG2 Cells Treated with Ferulic Acid

**Figure 1:**
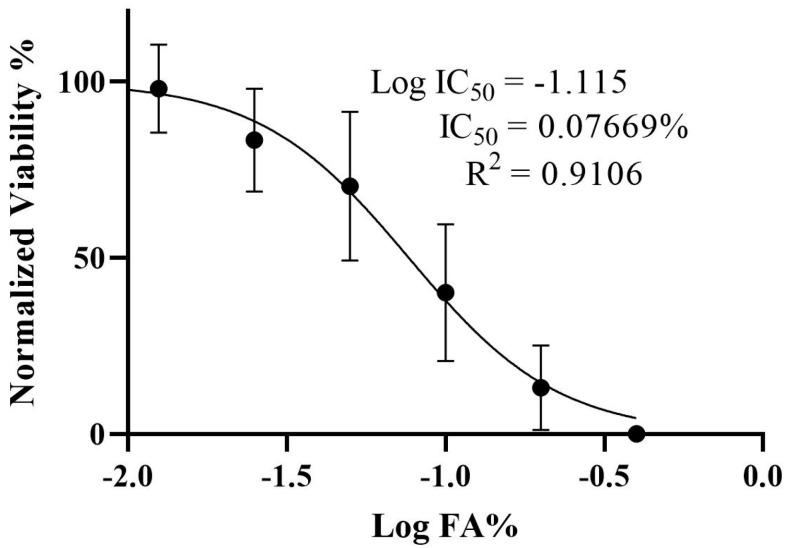
Determination of the IC_50_ and viability of HepG2 cells treated with ferulic acid. The viability percentage of viable HepG2 cells in response to treatment with different log percentages of ferulic acid was measured via MTT assay. Results represent M ± SD of independent triplicate experiments. IC_50_: Half maximum inhibitory concentration.

The effect of FA on HepG2 cells’ growth and proliferation was investigated using the MTT test. The results indicated an inverse relationship between viability and FA concentration; specifically, as the logarithmic concentration of FA increases, the cell viability decreases. Furthermore, the IC_50_ of FA was determined to be 0.07669% (Figure 1).

### 3.2. Effect of Ferulic Acid on Hepcidin Secretion and Expression

**Figure 2:**
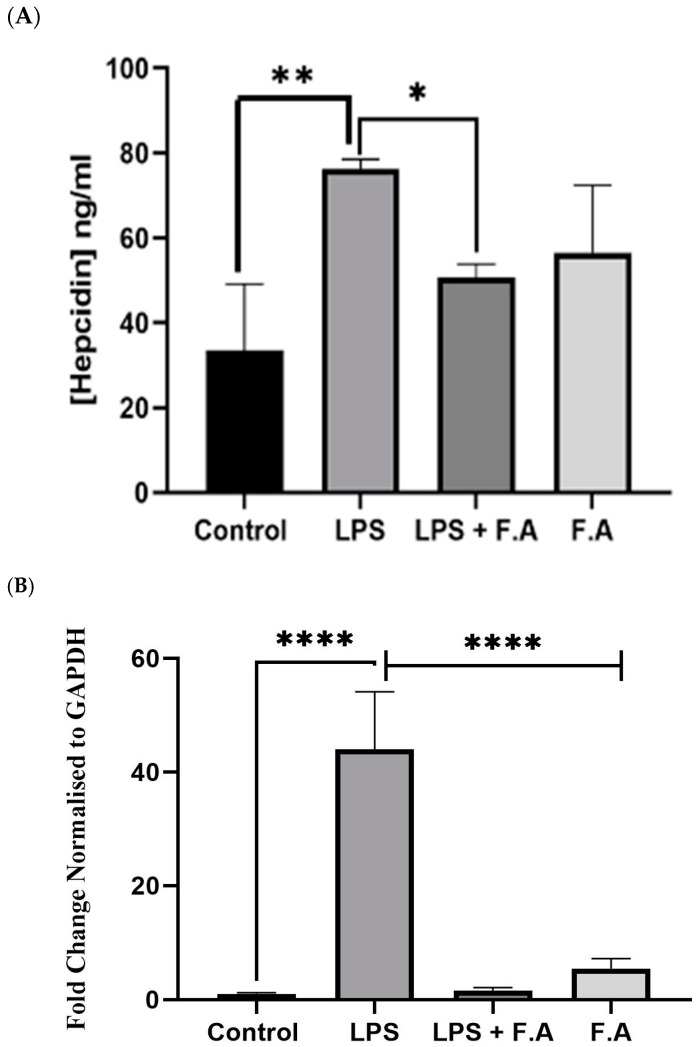
Reduction in hepcidin secretion and *HAMP* expression in response to ferulic acid. A 0.1% ferulic acid stock concentration diminishes (**A**) hepcidin secretion and (**B**) *HAMP* expression in HepG2 cells. Results represent the M ± SD of independent triplicate experiments. *p*-values indicate significance, as determined by Student’s paired *t*-test or the one-way ANOVA test. Significance levels are represented as follows: * *p* ≤ 0.05, ** *p* ≤ 0.01, **** *p* ≤ 0.0001.

In order to investigate the impact of FA on the secretion of hepcidin, we quantified the hepcidin levels induced by *IL-6* in HepG2 cells subjected to LPS treatment. This assessment was conducted following a 24 h exposure to 0.1% FA. A marked increase (*p* ≤ 0.01) in the hepcidin secretion was observed in LPS-treated cells compared to the controls. Also, a significant reduction (*p* ≤ 0.05) in the concentration of secreted hepcidin was observed in cells treated with 0.1% FA after they had been treated with LPS. Conversely, cells that received 4 μg/mL of LPS alone exhibited a marked elevation in hepcidin levels (*p* ≤ 0.05). Furthermore, the hepcidin secretion from HepG2 cells treated with 0.1% FA after incubation with 4 μg/mL LPS demonstrated a marked decline (Figure 2A). Figure 2B shows the influence of LPS on the modulation of hepcidin following FA exposure in HepG2 cells; it specifically analyzes *HAMP* expression levels in the presence of FA or LPS. Treatment with FA alone led to a slight increase in *HAMP* expression compared to the controls. Meanwhile, cells treated with LPS alone had significantly (*p* ≤ 0.0001) upregulated expression of the *HAMP* gene. However, the addition of 0.1% FA to the LPS-treated cells led to a significant (*p* ≤ 0.0001) approximately four-fold decrease in *HAMP* expression levels.

### 3.3. Effect of Ferulic Acid on IL-6 Expression and Secretion

**Figure 3:**
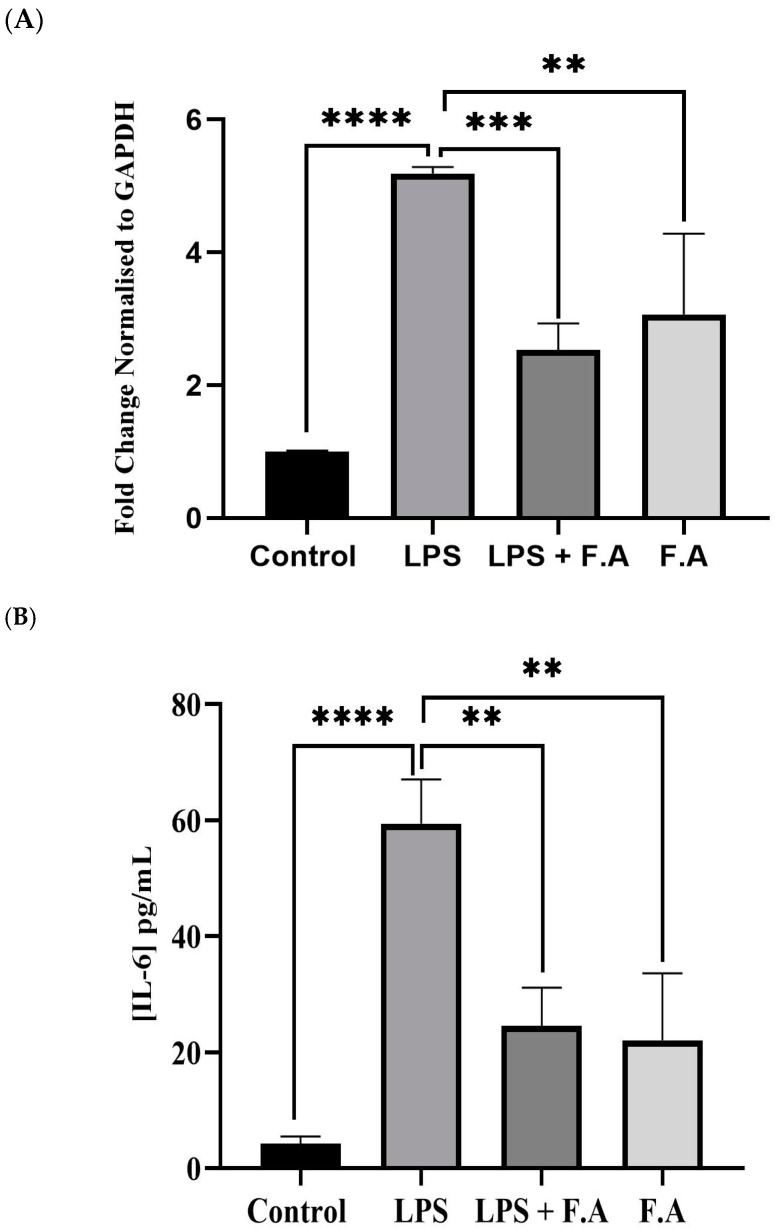
Reduction in proinflammatory *IL-6* expression and secretion via ferulic acid. A 0.1% of ferulic acid stock concentration decreased both the expression (**A**) and secretion of proinflammatory *IL-6* in HepG2 cells (**B**). Results represent the M ± SD of independent triplicate experiments. *p*-values indicate significance, as determined by the one-way ANOVA test. Significance levels are represented as follows: ** *p* ≤ 0.01, *** *p* ≤ 0.001, **** *p* ≤ 0.0001.

To measure the impact of LPS on *IL-6* levels after FA treatment in HepG2 cells, we looked at *IL-6* gene expression in response to treatment with either FA or LPS alone and their combination. The presence of 0.1% FA alone increased *IL-6* gene expression; however, this increase was significantly lower (*p* ≤ 0.01) than that in cells treated with 4 μg/mL LPS alone. The combination of 0.1% FA and LPS caused a significant (*p* ≤ 0.001) downregulation of *IL-6* gene expression compared to when cells were treated with LPS alone (Figure 3A). Additionally, to assess how FA affects inflammation in HepG2 cancer cells, we measured the increase in *IL-6* levels in cells treated with LPS for 24 h. Interestingly, *IL-6* levels showed a marked increase (*p* ≤ 0.0001) in cells treated with LPS alone compared to controls. Meanwhile, cells treated with both FA and LPS exhibited a significant reduction in *IL-6* levels (*p* ≤ 0.01). This reduction was about three times lower with FA than with LPS, as shown in Figure 3B.

### 3.4. Effect of Ferulic Acid on Phospho-STAT3 Secretion

**Figure 4:**
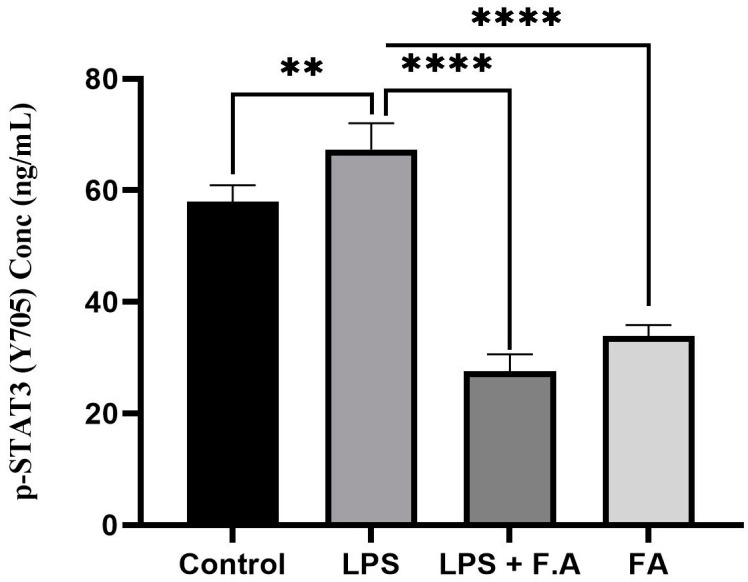
Reduction in phospho-STAT3 (Y-705) secretion in response to ferulic acid. A 0.1% ferulic acid stock concentration reduces secretion of phospho-STAT3 (Y-705) in HepG2 cells. Results represent the M ± SD of independent triplicate experiments. *p*-values indicate significance, as determined by one-way ANOVA test. Significance levels are represented as follows: ** *p* ≤ 0.01, **** *p* ≤ 0.0001.

To determine whether the modulation of hepcidin levels by ferulic acid occurred via the IL-STAT3 signaling cascade, we assessed phospho-STAT3 (Y-705) levels in HepG2 cells treated with FA alone, LPS alone or their combination. The results show that there was a significant (*p* ≤ 0.001) increase in phospho-STAT3 (Y-705) levels in response to LPS alone compared to the controls. Meanwhile, the administration of 0.1% FA alone or 1% FA after LPS treatment resulted a significant reduction in phospho-STAT3 (Y-705) levels (*p* ≤ 0.0001). However, the reduction in the phospho-STAT3 (Y-705) level in response to 1%FA after LPS induction was substantially larger than that when the cells were treated with FA alone, as illustrated in Figure 4.

## 4. Discussion

The importance of iron is not only simply restricted to its crucial role in hemoglobin synthesis and as an oxygen transporter throughout the body. It has fundamental importance extending to DNA synthesis and repair, cognitive development and immune system support [8,19]. Although iron deficiency is implicated in several health issues like anemia, excess iron levels can also lead to a serious health complications [20]. This highlights the importance of balanced consumption and meticulous oversight in medical environments.

Inflammatory environments can profoundly disrupt iron homeostasis, resulting in maladaptive iron metabolism that may aggravate disease mechanisms. Numerous studies have demonstrated the essential features of this interplay. For instance, Rosenblum, 2023 illustrated that the master regulator of iron metabolism, hepcidin, was induced by the proinflammatory cytokine *IL-6* and that this inducement leads to hypoferremia during the inflammation state [21]. This aligns with our study, which revealed that upon treating cells with LPS, there was an increase in *IL-6* gene expression and protein secretion, signifying a heightened state of inflammation (refer to Figure 3A,B). Furthermore, the expression of the hepcidin gene, along with its secretion, was notably upregulated and significantly elevated when the cell line was treated with LPS in this way. It was elucidated that this occurs via the STAT3 signaling pathway, emphasizing the pivotal role of *IL-6* and the interaction with its receptor, which results in the phosphorylation of STAT3 and the subsequent expression of hepcidin [22]. The findings of that research correlate with our results, as illustrated in Figure 2, Figure 3 and Figure 4.

The relationship between inflammatory responses and iron homeostasis is significant in numerous pathological states, such as cancer diseases and cardiovascular disorders [21]. Focusing on iron metabolism mechanisms offers prospective therapeutic avenues for the treatment of inflammatory disorders [23]. Thus, the aim of this study was to target iron metabolic pathways, particularly the STAT3 pathway, by using FA, which may present a potential alternate for therapeutic intervention in the treatment of these conditions.

FA is a multifaceted phenolic compound acknowledged for its broad spectrum of bioactive characteristics, rendering it advantageous across diverse domains [24]. Specifically, it demonstrates potential in the management of metabolic disorders through the mitigation of oxidative stress [25] and a protective role against the side effects of radiation therapy [26]. Cancer, as previously noted, is categorized as a metabolic disorder; consequently, FA was employed in this investigation as a prospective protective or therapeutic alternative for cancer treatment. Our study revealed that the *IL-6* concentration was approximately threefold greater in cells treated with LPS compared to those receiving FA treatment after LPS stimulation. Nonetheless, the use of FA effectively mitigated the elevation of *IL-6* levels induced by LPS exposure. Specifically, the application of FA (0.1%) conferred protective effects against the inflammatory response elicited by LPS, resulting in a reduction in *IL-6* secretion to nearly one-third of the levels recorded in cells treated exclusively with LPS (Figure 3A). The investigation reported in [27] aligns with our research findings, i.e., using FA effectively reduces the LPS-mediated increase in *IL-6*. In particular, FA (0.1%) helped protect against the inflammatory reaction caused by LPS, lowering *IL-6* secretion to nearly one-third of what was seen in cells treated only with LPS (refer to Figure 3). In response to this reduction, the hepcidin level was also reduced, supporting the hypothesis that FA may confer a protective effect against the *IL-6*-mediated responses elicited by LPS, as shown in Figure 2 and Figure 3. Curcumin, a natural compound, along with AG490 and a peptide that diminishes hepcidin expression in hepatocytes, together obstruct the *IL-6*-STAT3 signaling pathway and may present an innovative strategy for addressing iron dysregulation in the context of inflammatory diseases [27]. Another separate investigation undertaken by Li et al., 2022 corelates with our findings. In their study, it was demonstrated that caffeine attenuates hepcidin expression through the suppression of inflammation and the modulation of the *IL-6*/STAT3 signaling pathway [22], thereby presenting a compelling potential intervention for the management of anemia associated with inflammation. A study by Xin et al., 2016, showed that hydrogen sulfide reduces hepcidin production through the *IL-6*/STAT3 pathway; this also agrees with our findings [28]. Consequently, the correlation between the inflammatory milieu instigated by LPS stimulation, which leads to a reduction in hepcidin levels, has been empirically demonstrated to occur via the IL-STAT3 signaling cascade, as evidenced by our findings, which showed that FA treatment mitigated the rise in STAT3 levels subsequent to LPS stimulation in the HepG2 cell lines.

Conversely, FA not only confers a protective effect but also exhibits a cytotoxic effect against HepG2 cell lines, as demonstrated in Figure 1. This finding corelates with the observations made by the authors of [29], who indicated that FA enhances apoptosis in renal carcinoma (ACHN) and cervical carcinoma (HeLa and Caski) cell lines, with IC_50_ values recorded at 30 μM and 4–20 µM, respectively. Although the cytotoxic properties of FA appear promising, certain investigations suggest diminished efficacy at elevated concentrations in specific cancer cell lines, indicating that additional research is warranted to comprehensively elucidate its therapeutic potential.

FA has been demonstrated to be a potential therapeutic agent in vitro; however; its progress in this context may be impeded due to its inadequate solubility and bioavailability and the complex nature of its metabolic pathways. These factors represent a considerable obstacle to its utilization as a therapeutic agent, as the absorption and distribution of FA within the in vivo system can be adversely affected [30]. Initiatives aimed at ameliorating these characteristics include the formulation of FA nanoemulsions and derivatives, which have led to improved solubility and bioavailability in vitro [31]. Additionally, synthesizing innovative derivatives of FA with enhanced physicochemical attributes has exhibited potential to bolster its therapeutic capacity [30]. Another limitation that remains unexplored in the current study is the intricate metabolic transformation of FA within the gut microbiome, where its metabolites exert diverse effects on microbial populations. This complexity has the potential to affect the therapeutic outcomes of using FA [32]. Although FA displays promise as a therapeutic agent, these limitations highlight the need for the development of additional future research targets’ development to optimize its effectiveness in in vivo systems, i.e., via simulating an animal model of inflammation. Moreover, a co-culture of HepG2 and Caco-2 cell lines will allow a chance at the investigation of FA’s effect on iron levels, other potential regulatory factors of *HAMP* expression like IL-1β and activin-B, ferroportin levels and molecular functional studies. Furthermore, future studies are needed to investigate the effect of FA on other cellular pathways such as activation of NF-κB and MAPK.

## 5. Conclusions

We have shown, for the first time, that FA significantly reduces hepcidin expression and secretion levels through attenuation of the *IL-6*-STAT3 pathway; hepcidin is one of the key regulators of iron metabolism. Thus, this collectively underscores the intricate interplay between FA and inflammatory mediators, thereby elucidating the potential therapeutic and protection implications of FA in the management of conditions characterized by dysregulated hepcidin levels such as cancer, anemia and chronic inflammation.

## Figures and Tables

**Table 1:** Target gene primer sequences for quantitative real-time polymerase chain reaction.

Target Gene	Forward (3′ to 5′)	Reverse (3′ to 5′)
*HAMP*	TTCCTTCCTGGGCATGGA GT	GCAATGATCTTGATCTTCATT
*IL-6*	CACAACAGACGGGACAACTT	CGCAGCAGAAAATGCAGATG
*GAPDH*	GCCAAAAGGGTCATCATCTC	GGTGCTAAGCAGTTGGTGGT

## Data Availability

All the data are presented within the paper.

## Abbreviations

The following abbreviations are used in this manuscript:

FA	Ferulic acid
HCC	Hepatocellular carcinoma
DMSO	Dimethyl sulfoxide
ACHN	Apoptosis in renal carcinoma
